# Impact of the COVID-19 pandemic on the pattern of acute appendicitis, a single-center study

**DOI:** 10.3389/fsurg.2026.1778112

**Published:** 2026-05-15

**Authors:** Khaled A. Obeidat, Rami A. Saadeh, Renad Y. Msameh

**Affiliations:** 1 Department of Surgery, Faculty of Medicine, Jordan University of Science and Technology, Irbid, Jordan; 2 Department of Public Health, Faculty of Medicine, Jordan University of Science and Technology, Irbid, Jordan; 3 Department of Surgery, King Abdullah University Hospital, Irbid, Jordan

**Keywords:** acute appendicitis, appendectomy, complicated appendicitis, COVID-19, perforation

## Abstract

**Background:**

Acute appendicitis is a common surgical emergency. During the COVID-19 pandemic, a decline in emergency department visits for various conditions, including appendicitis, was noted.

**Objective:**

To evaluate changes in the number, clinical presentation, and surgical approach of appendectomies performed during the COVID-19 lockdown compared to a pre-pandemic period.

**Design:**

Retrospective cohort study.

**Patients:**

All patients who underwent appendectomy for acute appendicitis during the lockdown months (March–May 2020 and 2021) were compared to those who had appendectomy during the same months in 2018–2019.

**Main outcome measures:**

The rates of complicated (perforated) appendicitis, total number of procedures, and surgical modality (open vs. laparoscopic) were compared between the two periods.

**Results:**

A total of 252 patients were included. During the pandemic period, 19.4% of patients presented with a perforated appendix vs. 9.3% in the pre-pandemic group (*p* = 0.045, OR = 2.46). A significant decrease in the total number of appendectomies was observed during the pandemic (*p* < 0.001). Additionally, the proportion of open appendectomies increased during the lockdown.

**Conclusion:**

In our study, the COVID-19 lockdown was associated with a significant increase in the rate of perforated appendicitis, a reduction in hospital-treated cases, and a higher rate of open surgery. These findings may suggest delayed patient presentation during the pandemic or a relative decrease in less severe cases.

## Introduction

Coronavirus disease 2019 (COVID-19) emerged in late 2019 as a global outbreak caused by the novel severe acute respiratory syndrome coronavirus 2 (SARS-CoV-2) first emerged in late 2019. Since its initial identification, COVID-19 has posed an unprecedented challenge to healthcare systems worldwide. As of February 2026, more than 779 million confirmed cases and over 7 million deaths have been reported to the World Health Organization (WHO) (1).

In January 2020, the WHO declared the outbreak a Public Health Emergency of International Concern. Subsequently, on March 11, 2020, it characterized COVID-19 as a global pandemic (2).

Governments implemented various strategies to curb the spread of COVID-19. Many countries imposed lockdowns, instructing the public to remain at home and minimize contact with others to reduce both infection with and transmission of the virus. Non-emergent surgical procedures were postponed, and outpatient clinics were temporarily suspended to ensure that hospital resources were available for patients with COVID-19 and to further limit disease transmission (3). In Jordan, the first confirmed case of coronavirus disease 2019 (COVID-19) was officially reported on 2 March 2020 (4). In response, the government implemented a nationwide lockdown that remained in effect for three months, from March through the end of May 2020. In 2021, a similar lockdown was reimposed during the same period and for an equivalent duration following a resurgence in COVID-19 cases (3).

During the COVID-19 pandemic, emergency departments were primarily focused on managing infected patients; however, acute surgical pathologies continued to present and required timely evaluation and intervention. At our institution, a significant reduction in overall emergency department visits was observed during the acute lockdown period compared with corresponding periods in previous years.

Acute appendicitis is the most common general surgical emergency, accounting for 4.5%–23% of presentations with abdominal pain to the emergency department (5, 6). Appendectomy remains the standard of care (7, 8). Delayed diagnosis or treatment may result in disease progression to complicated appendicitis, defined by the presence of gangrene, abscess formation, or perforation (9–11).

Reported perforation rates range from 2% to 40%, with higher rates observed in pediatric populations and in patients older than 50 years (12–14). Perforated appendicitis is associated with substantially increased morbidity and mortality compared with uncomplicated disease, with mortality rates reported up to 5% in perforated cases (12).

During the COVID-19 pandemic, delayed presentation to medical care was frequently reported and has been recognized as a risk factor for complicated appendicitis (15, 16).

The present study aimed to study the characteristics of surgically managed appendicitis during COVID-19 lockdown, including the timing of hospital presentation, overall number of appendectomies, and the operative management strategy at our institution.

## Methods

This study was a retrospective cohort investigation of acute appendicitis conducted at King Abdullah University Hospital, a tertiary care academic medical center in northern Jordan.

After obtaining approval from the Institutional Review Board of Jordan University of Science and Technology and King Abdullah University Hospital, all patients who underwent surgery for a provisional diagnosis of acute appendicitis during March, April, and May of 2020 and 2021 (post-pandemic group) were identified. These months were selected because they corresponded to the nationwide lockdown imposed by the Jordanian government in response to the COVID-19 pandemic.

For comparison, all patients who underwent appendectomy for acute appendicitis during the same months in 2018 and 2019 were identified as the pre-pandemic group. The patients were identified from the surgical logs. Patients who underwent appendectomy for indications other than a provisional diagnosis of acute appendicitis and those with missing data were excluded.

Complicated appendicitis was defined as the presence of appendiceal perforation, gangrenous appendicitis, and/or abscess or phlegmon formation. No cases of acute appendicitis managed conservatively could be identified during the study periods. All data were retrieved from the hospital's electronic medical records.

The two groups were compared to assess differences in the number of patients undergoing appendectomy for acute appendicitis before and after the COVID-19 outbreak, as well as to determine whether there was an increase in the proportion of patients presenting with complicated appendicitis.

### Data collection

The variables collected included patient age, sex, duration of symptoms prior to presentation to the emergency department, preoperative symptoms and signs, imaging findings, and laboratory results. Operative findings, length of hospital stay, postoperative complications, and histopathological findings were also recorded.

### Statistical analysis

Descriptive statistics were used to summarize patient demographic characteristics, clinical presentation, operative findings, and postoperative complications. The chi-square test was used for categorical variables and the student's *t*-test for continuous variables to examine differences in the frequency distribution of different variables between the COVID-19 period (2020–2021) and the pre-pandemic period (2018–2019). Missing variables were excluded from the analysis, and the odds ratio was calculated using a 2 × 2 contingency table. Multivariable analysis was explored, but didn't identify independent predictors. All statistical analyses were performed using IBM SPSS Statistics software, version 26. A *p*-value of <0.05 was considered statistically significant.

## Results

A total of 252 patients who were admitted with acute appendicitis and underwent appendectomy during the study period were identified. The overall cohort comprised 144 males (57.1%) and 108 females (42.9%). There were no statistically significant differences between the two groups with respect to age and sex distribution. Of the total, 176 patients (69.8%) were in the pre-pandemic group (2018–2019) and 76 patients (30.2%) were in the post-pandemic group (2020–2021). This represents a statistically significant decrease of more than 50% in the number of patients operated on for acute appendicitis (*p* < 0.001) (Table 1).

**Table 1:** Comparison of cases during the 2 years of COVID-19 and previous years.

Variable	2018 and 2019	2020 and 2021	*p*-value
Age (years)	24.51 ± 2.23	23.63 ± 3.28	0.67
Sex			
Female	80 (45%)	28 (37%)	0.19
Male	96 (55%)	48 (63%)	
Number of cases	176 (69.8)	76 (30.2)	<0.001
Duration of symptoms (hours)			
*Less than 36 h*	58.2%	49.2%	0.32
*Equal or More than 36 h*	41.8%	50.8%	
RLQ Tenderness			
*Positive*	97.1%	94.1%	0.28
*Negative*	2.9%	5.9%	
Temperature			
*Less than 37.5*	75.5%	76.2%	0.912
*More than 37.5*	24.5%	23.8%	
WBC			
*4.5–11*	26.5%	23.4%	0.734
*More than 11*	73.5%	76.6%	
CT Acute Appendicitis			
*Positive*	65.5%	61.3%	0.692
*Negative*	34.5%	38.7%	
CT Perforation			
*Positive*	13.8%	23.3%	0.219
*Negative*	86.2%	76.7%	
Operative findings—Perforation			
*Positive*	9.3%	19.4%	0.045
*Negative*	90.7%	80.6%	
Hospital Stay			
4 days or less	80.0%	85.5%	0.351
More than 4 days	20.0%	14.5%	
Wound infection			
*Positive*	2.3%	5.3%	0.248
*Negative*	97.7%	94.7%	

Operative findings demonstrated a significantly higher rate of appendiceal perforation in the post-pandemic cohort compared with the pre-pandemic cohort (19.4% vs. 9.3%, *p* = 0.045). This association was further quantified by calculating the odds ratio. Patients in the post-pandemic group had 2.46-fold higher odds of intraoperative perforation [odds ratio (OR) 2.46; 95% confidence interval (CI): 1.14–5.27] compared with those in the pre-pandemic group.

The mean duration of symptoms before presenting to the hospital was longer in the post-pandemic group compared to the pre-pandemic group (41.3 ± 8.27 h vs. 34.3 ± 4.56 h). However, this difference was not statistically significant (*p* = 0.126).

The utilization of CT imaging for the diagnosis of acute appendicitis increased during the post-pandemic period (69%) compared to the pre-pandemic period (58%). Furthermore, findings of perforated appendicitis on CT were more frequent in the post-pandemic group, though this difference was not statistically significant (23.3% vs. 13.8%, *p* = 0.219).

There was also a significant change in the surgical approach. During the initial post-pandemic period (2020), there was a marked increase in the rate of open appendectomy compared to the pre-pandemic period. Open appendectomies accounted for 62.5% of cases in 2020 vs. 37.5% for laparoscopic, a significant shift from the pre-pandemic practice (18% open vs. 82% laparoscopic, *p* = 0.01). In 2021, laparoscopic appendectomy returned to the pre-pandemic pattern.

The rate of postoperative wound infections, while higher in the post-pandemic group (5.3% vs. 2.3%), did not reach statistical significance (*p* = 0.248).

No statistically significant differences were observed between the two groups regarding preoperative clinical findings (including RLQ tenderness, temperature, and WBC count), hospital length of stay, or other pathological findings.

## Discussion

During the government-imposed lockdowns implemented in 2020 in response to the COVID-19 pandemic and to limit the spread of SARS-CoV-2, a marked reduction in emergency department visits was observed both at our center and worldwide (17–19). This decline extended to non-communicable conditions, including acute appendicitis, for which a significant reduction in presentations was reported locally and internationally (20, 21). One possible explanation for the decrease in hospital-treated cases of acute appendicitis is that milder cases may have been managed conservatively in the community or may have resolved spontaneously without hospital referral.

Public fear of contracting COVID-19, together with governmental recommendations to remain at home and minimize hospital visits, may also have contributed to delayed presentation to healthcare facilities. Furthermore, several reports have demonstrated that multiple categories of urgent surgical conditions were affected during the pandemic period (22).

A delay in presentation is a critical prognostic factor in acute appendicitis, as it is associated with a higher risk of progression to perforation (23, 24). It was therefore foreseeable that the pandemic period would be characterized by more advanced disease pathology at the time of diagnosis.

Our study observed a longer mean duration of symptoms in the post-pandemic group, suggesting a trend toward delayed presentation. However, this difference did not reach statistical significance, a finding that may be attributable to the limited sample size. This places our results within a broader context of conflicting reports in the literature. While some authors have reported a significant delay in presentation during COVID-19-related restrictions (15, 16, 25), others, consistent with our findings, found no such significant delay (26, 27).

The clinical significance of any delay is underscored by its potential to increase the incidence of complicated appendicitis (9–11). An increased rate of complicated appendicitis in the post-pandemic period has been reported in several other reports (15, 16, 25, 28). However, the underlying cause of this observed increase is debated. While some attribute it directly to delayed presentation (15, 16, 25, 28), Andersson et al., in their meta-analysis, proposed an alternative explanation: the increased proportion of complicated cases may be driven by a relative decrease in uncomplicated appendicitis, which were more likely to be managed non-operatively with antibiotics or to resolve spontaneously without hospital referral (29). This hypothesis is supported by Neufeld et al., who noted a significant decline in uncomplicated appendicitis during the pandemic, with no corresponding decrease in complicated cases (30).

The clinical implications of appendiceal perforation are substantial. Perforated appendicitis is associated with significantly higher morbidity and mortality compared with non-perforated disease, with mortality rates reported to reach up to 5% in some series (12, 31). Additionally, perforation is linked to prolonged hospital stay and increased readmission rates, thereby increasing overall healthcare costs (31, 32).

The diagnosis of appendicitis traditionally relies on clinical history, physical examination, and laboratory parameters, with cross-sectional imaging reserved for equivocal cases (12). During the pandemic, however, a shift in this paradigm was observed. The use of computed tomography (CT) imaging increased significantly in many centers (25, 33). This was driven by the need for rapid diagnostic confirmation to minimize unnecessary operations—and thus, patient and healthcare worker exposure to the risks of aerosol-generating procedures in a high-risk COVID-19 environment. In our study, we observed an increase in the utilization of CT imaging in the post-pandemic group. In addition, the finding of perforation on CT imaging was not increased despite the increased rate of perforation at operation. This comes as no surprise because studies have shown the CT to be less sensitive than specific for the detection of appendiceal perforation (34, 35).

Surgical appendectomy remains the standard treatment for acute appendicitis, with the laparoscopic approach considered the gold standard (12, 36). Laparoscopic surgery is associated with shorter hospital stay and lower rates of postoperative complications, including wound infection and intra-abdominal abscess formation, compared with open surgery (36, 37). During the early phase of the pandemic, open appendectomy was recommended in some guidelines to reduce potential exposure of healthcare workers to viral particles present in peritoneal fluid and aerosolized during pneumoperitoneum release (38), particularly in centers lacking closed-circuit filtration systems, as was the case in our institution.

Reflecting these guidelines, our center observed a marked increase in the rate of open appendectomy during the first pandemic wave in 2020 (62.5% open vs. 37.5% laparoscopic). By 2021, surgical practice had reverted to the pre-pandemic pattern, with laparoscopy again predominating (82% laparoscopic vs. 18% open). This trend, with an increased proportion of open appendectomies during the initial pandemic phase, has been documented in several other studies (21, 25, 26, 29).

Regarding postoperative wound infection, we observed a higher incidence during the post-pandemic period; however, the difference was not statistically significant. The literature remains inconclusive on this issue, with some studies reporting increased wound infection rates during the pandemic (39, 40), while others have found no significant difference (41, 42).

## Conclusion

In summary, our study demonstrates a significant increase in intraoperative findings of perforation among patients with acute appendicitis in the post-pandemic period compared to the pre-pandemic cohort. Furthermore, we observed a marked decline in the overall number of hospital-treated cases of acute appendicitis during the post-pandemic era, accompanied by a shift toward open appendectomy during the initial wave of the COVID-19 pandemic in 2020.

### Limitations

This study has limitations. First, its retrospective design and single-center setting limit the generalizability of the findings and resulted in a relatively small sample size. Second, we were unable to identify patients with acute appendicitis who were managed conservatively without surgical intervention, which may have influenced the overall case distribution and outcome assessment.

## Data Availability

The raw data supporting the conclusions of this article will be made available by the authors, without undue reservation.
